# Development of a Class-C Power Amplifier with Diode Expander Architecture for Point-of-Care Ultrasound Systems

**DOI:** 10.3390/mi10100697

**Published:** 2019-10-14

**Authors:** Hojong Choi

**Affiliations:** Department of Medical IT Convergence Engineering, Kumoh National Institute of Technology, Gumi 39253, Korea; hojongch@kumoh.ac.kr; Tel.: +82-54-478-7782

**Keywords:** class-C power amplifier, diode expander, piezoelectric transducers, point-of-care ultrasound systems

## Abstract

Point-of-care ultrasound systems are widely used in ambulances and emergency rooms. However, the excessive heat generated from ultrasound transmitters has an impact on the implementation of piezoelectric transducer elements and on battery consumption, thereby affecting the system’s sensitivity and resolution. Non-linear power amplifiers, such as class-C amplifiers, could substitute linear power amplifiers, such as class-A amplifiers, which are currently used in point-of-care ultrasound systems. However, class-C power amplifiers generate less output power, resulting in a reduction of system sensitivity. To overcome this issue, we propose a new diode expander architecture dedicated to power amplifiers to reduce the effects of sinusoidal pulses toward the power supply. Thus, the proposed architecture could increase the input pulse amplitudes applied to the main transistors in the power amplifiers, hence increasing the output voltage of such amplifiers. To verify the proposed concept, pulse-echo responses from an ultrasonic transducer were tested with the developed class-C power amplifier using a resistor divider and the designed diode expander architecture. The peak-to-peak amplitude of the echo signals of the ultrasonic transducers when using a class-C power amplifier with a diode expander architecture (2.98 V_p–p_) was higher than that for the class-C power amplifier with a resistor divider architecture (2.51 V_p–p_). Therefore, the proposed class-C power amplifier with diode expander architecture is a potential candidate for improving the sensitivity performance of piezoelectric transducers for point-of-care ultrasound systems.

## 1. Introduction

Ultrasound has been widely used for patient diagnosis because it is a non-ionizing, real-time, non-invasive, and inexpensive imaging modality [1]. Especially, point-of-care ultrasound systems have been used to diagnose acute diseases for immediate treatment in ambulance vehicles and emergency rooms with remote help from physicians [2,3]. However, the performance of point-of-care ultrasound systems typically suffers from a limited number of ultrasonic array transducer elements, overheating, and battery issues, thereby affecting the sensitivity and resolution of such ultrasound systems [3,4]. To overcome overheating and battery issues, manufacturers need to use cooling systems such as cooling fans and aluminum heat pipe structures even though the internal structures of point-of-care ultrasound systems are much smaller than conventional bench-top ultrasound machines [3].

Conventional ultrasound systems are composed of ultrasound transmitters, ultrasonic (piezoelectric) transducers, and ultrasound receivers [5,6,7]. Most of the overheating issues come from the power amplifiers in the ultrasound transmitters and by the analog-to-digital converter or digital-to-analog converter in the ultrasound receivers [8]. However, the excessive heat could be resolved by employing non-linear power amplifiers in the ultrasound transmitters owing to their different transition period between voltage and current [9]. Among non-linear power amplifiers, class-C power amplifiers could achieve relatively low DC current consumption while lowering the gain [10]. Therefore, the scheme to increase the gain would be beneficial for class-C power amplifiers in low-sensitivity ultrasonic transducers.

Several non-linear power amplifiers have been developed using different types of ultrasonic transducers to improve the performance of ultrasound instruments. A class-E inverter was proposed for inductive piezoelectric transducers to determine the optimal resonance frequency based on the equivalent circuit model [11]. A class-D half-bridge driving power amplifier was proposed for piezoelectric transducers to find the optimal conversion frequency operation [12]. A class-E power amplifier was proposed to reduce power dissipation in Langevin transducers [13]. 

**Figure 1:**
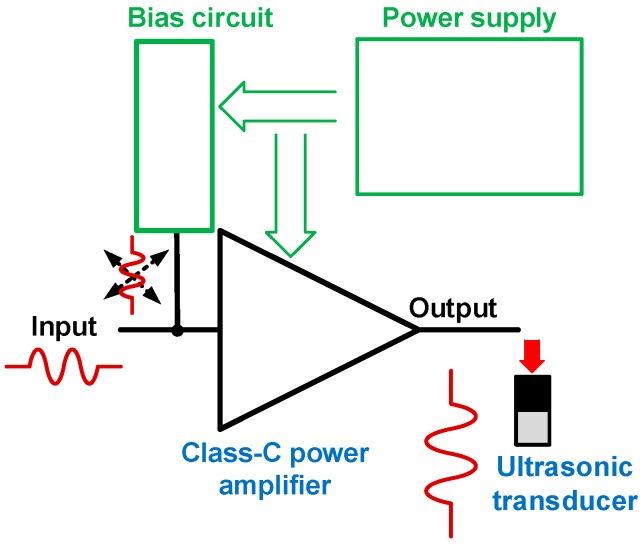
Concept for class-C power amplifiers with input pulse signals and bias circuit.

Figure 1 describes how input signals in power amplifiers affect the bias circuit and how the power supply controls the power amplifiers through the bias circuit. Positive and negative pulse signals can pass through the bias circuit and get into the power supply while the power supply is applied to DC voltages through the bias circuit to the power amplifier [10]. In the output, the amplified positive and negative pulse signals trigger the ultrasonic transducer. The bias circuit needs to be stabilized during signal amplification through control of the power supply. Therefore, the pulse signals should not affect the bias circuit operation. Otherwise, it could generate output signals with high noise and harmonic distortion or reduce the output signal amplitudes. These scenarios directly affect the resolution and sensitivity performance of ultrasound systems [2,14]. 

To reduce the effects of pulse signals on bias circuits, different architecture using an analog-to-digital converter, a digital-to-analog converter, and a look-up table have been used for wireless applications [15]. This method needs to regulate several DC bias voltages to properly operate the transistors. This might not be suitable for point-of-care ultrasound systems because of their higher DC current consumption. Additionally, a number of ultrasonic transducers are required for combination with each transmitter channel to achieve a limited size of point-of-care ultrasound systems. However, the proposed method is a much simpler approach for easy regulation of a single power amplifier, thereby increasing the output voltages of the power amplifiers for point-of-care ultrasound systems.7

A power metal-oxide semiconductor field-effect transistor (MOSFET) simulation model has been developed to estimate the expected performance in a high-voltage environment [9]. Given that a power MOSFET simulation library for a high-voltage environment provides inaccurate performance for sub-decibel level operation, the performance of power amplifiers needs to be verified through real measurements [15,16]. Therefore, in Section 2, we report the detailed experimental circuit design analysis in DC and AC levels rather than calculated and simulated results. The equivalent circuit model with measured parameters for power amplifiers is provided. Section 3 shows the measured performance results for the power amplifier including pulse-echo response with ultrasonic transducers. Section 4 concludes the paper.

## 2. Materials and Methods 

Diode expanders placed after power amplifiers have originally been used to reduce unwanted ring down signals generated from class-A power amplifiers in ultrasound instruments. Class-A amplifiers produce a higher gain compared to class-C power amplifiers while consuming a higher DC current. Therefore, class-A power amplifiers can use relatively lower input voltage signals compared to class-C power amplifiers. In ultrasound instruments, the maximum allowable input voltages are generally limited to 1 V_p–p_ [17,18]. Therefore, the bias circuits in class-A power amplifiers would be less affected by input pulse signals. However, class-C power amplifiers produce a relatively lower voltage gain compared to class-A power amplifiers and also consume a lower DC current. Consequently, higher input voltage signals could affect the bias circuit operation for class-C power amplifiers. Overall, a diode expander architecture is proposed for bias circuits of class-C power amplifiers because higher input pulse signals can pass through the bias circuits while the power supply controls the bias circuits and the amplified pulse signal reaches the ultrasonic transducers. The proposed diode expander architecture is intended to suppress unwanted input pulse signals into the bias circuits, resulting in stabilization of the DC current and improvement of the output voltages of class-C power amplifiers. 

**Figure 2:**
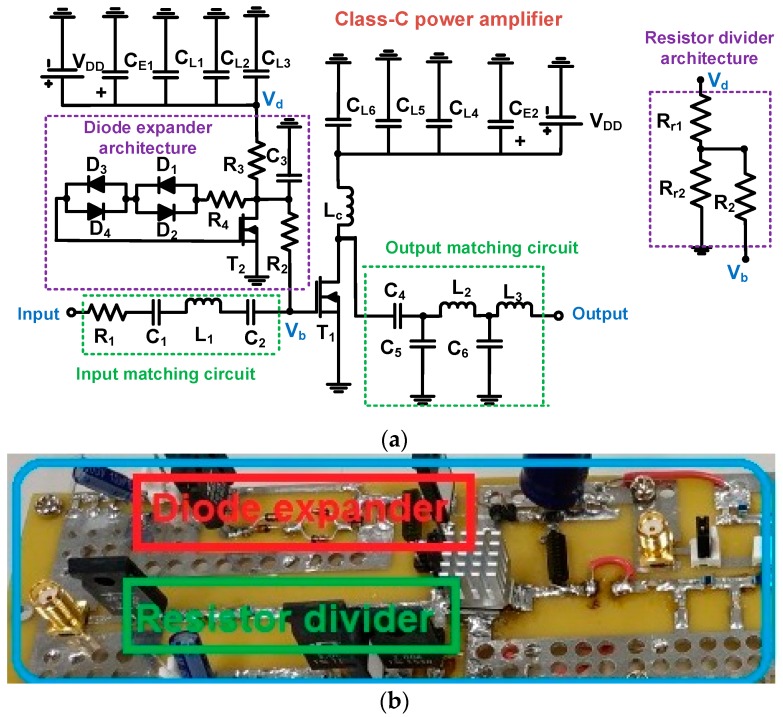
(**a**) Schematic diagram and (**b**) implemented printed circuit board of the developed class-C power amplifier with a resistor divider and diode expander architecture.

Figure 2 shows a schematic diagram and implemented printed circuit board of the developed class-C power amplifier with a resistor divider and with a diode expander architecture. The printed circuit board was fabricated from a manufacturing service (ExpressPCB LLC, Mulino, OR, USA). The mounting holes were tied to reduce parasitic effects. The signal traces were not perpendicularly cross the ground plane. Given that high-voltage pulse signals are needed to trigger ultrasonic transducers, power film resistors, capacitors, inductors, choke inductors, and power transistors are usually selected because they tolerate high voltages. The selected power transistors (T_1_, PD57018-E, STMicroelectronics Inc., Geneva, Switzerland) were lateral-diffusion metal-oxide semiconductor field-effect transistors (LDMOSFETs) that can be used for high voltages up to 65 V and high currents up to 2.5 A operations. A small-size (1 cm length and width) heat-sink was attached on the top of the LDMOSFET devices. Electrolytic capacitors (C_E1_ = 10 μF and C_E2_ = 220 μF) including additional ceramic capacitors (C_L1_ = 0.1 μF, C_L2_ = 1 nF, and C_L3_ = 47 pF) admitting up to 200 V were used to stabilize the DC voltages from the power supplies. A radio frequency (RF) choke inductor with a self-resonant frequency at 190 MHz (L_c_ = 1 μH) admitting high currents up to 2 A was selected. The power film resistors (R_1_ to R_5_) operated up to 250 V. The input and output matching circuits were used in the input and output sides, respectively, to match the operating frequency of the power amplifier. In the input matching circuit, one resistor, two capacitors, and one inductor were used. In the output matching circuit, three capacitors and two inductors were employed. In both matching circuits, the resistors, capacitors, and inductors also admitted high voltages, up to 100 V. Cooling fans were used for accurate measurement, though class-C power amplifiers generate less heat than class-A power amplifiers. Table 1 provides component values for the power amplifiers. 

**Figure 3:**
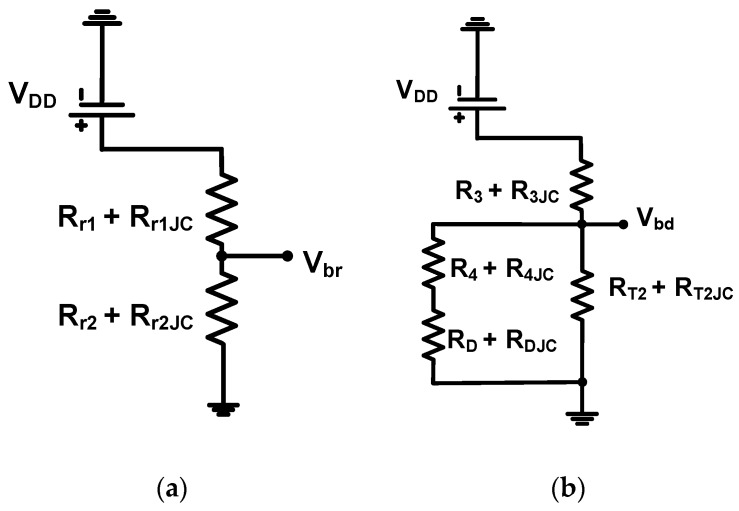
DC Operating mechanisms of the (**a**) resistor divider and (**b**) diode expander architecture.

Figure 3 shows the operating mechanisms of the resistor divider and diode expander architecture including thermal resistances. Although the manufacturers did not provide the thermal model parameters of the power film resistors, diodes, and LDMOSFETs, their thermal resistances were included to estimate thermal effects in the resistor divider and diode expander architecture. In the resistor divide architecture, the DC bias voltages were obtained by the ratio of two power film resistors (R_r1_ = 5 kΩ and R_r2_ = 500 Ω) with their corresponding thermal resistances (R_r1JC_ and R_r2JC_). In the diode expander architecture, the DC bias voltages were obtained by two power film resistors, diode equivalent resistance (R_D_), and drain-source resistance of one of the LDMOSFET (T_2_) including their thermal resistances (R_3JC_, R_4JC_, R_DJC_, and R_T2JC_).

In Figure 3a, the resistor divider architecture used two power film resistors (R_r1_ and R_r2_). The bias voltage of the resistor divider (V_br_) is expressed as:

(1) $$V_{\mathrm{br}}=\frac{R_{r2}\;+{\;R}_{r2\mathrm{JC}}}{R_{r1}\;+{\;R}_{r1\mathrm{JC}}+R_{r2}\;+{\;R}_{r2\mathrm{JC}}}V_{\mathrm{dd}}=\frac{1}{1+\frac{R_{r1}\;+{\;R}_{r1\mathrm{JC}}}{R_{r2}\;+{\;R}_{r2\mathrm{JC}}}}V_{\mathrm{dd}}$$

Here, V_dd_ is the supply voltage from the DC power supply and R_r1JC_, R_r2JC_, and R_r3JC_ are the thermal resistances of the power film resistors (R_r1_, R_r2_, and R_r3_, respectively).

The power MOSFET equivalent circuit model has been used to estimate the performance of diode expander architecture [19,20]. The diode expander architecture in Figure 3b used double cross-coupled diodes. The bias voltage of this architecture (V_bias2_) is expressed as:

(2) $$V_{\mathrm{bd}}=\frac{1}{1+\frac{R_{3}\;+{\;R}_{3\mathrm{JC}}}{\left(R_{T2}\;+{\;R}_{T2\mathrm{JC}}\right)//(R_{4}\;+{\;R}_{4\mathrm{JC}}+R_{D}\;+{\;R}_{\mathrm{DJC}})}}V_{\mathrm{dd}}\approx \frac{1}{1+\frac{R_{3}\;+{\;R}_{3\mathrm{JC}}}{R_{T2}\;+{\;R}_{T2\mathrm{JC}}}}V_{\mathrm{dd}}$$

where R_3JC_, R_T2JC_, R_4JC_, and R_DJC_ are the thermal resistances of the power film resistors (R_3_ and R_4_), the diode equivalent resistance (R_D_), and the drain-source resistance of LDMOSFET T_2_.

The thermal effect on the bias voltages for the resistor divider architecture is dependent on the two power film resistors (R_r1_ and R_r2_) with their corresponding thermal resistances (R_r1JC_ and R_r2JC_). Conversely, the thermal effect on the bias voltages for the diode expander architecture is dependent on a single power film resistor (R_3_) and the drain-source resistance of LDMOSFET R_T2_, with their corresponding thermal resistances (R_3JC_ and R_T2JC_). Therefore, the drain-source resistance of LDMOSFET R_T2_ is less insensitive to sudden temperature changes compared to power film resistors because temperature changes are dependent on resistance values [21]. To confirm these temperature dependences, the bias voltages for each architecture were measured after 1 min and 1 hr. At 1 min, the measured bias voltage of the class-C power amplifier when using the resistor divider and diode expander architecture was the same, i.e., 2.3 V. After 1 h, the measured bias voltage of the class-C power amplifier with the resistor divider and diode expander architecture did not coincide anymore; they were 2.25 V and 2.29 V, respectively. Therefore, we confirm that class-C power amplifiers with diode expander architecture are less dependent on temperature variations.

**Figure 4:**
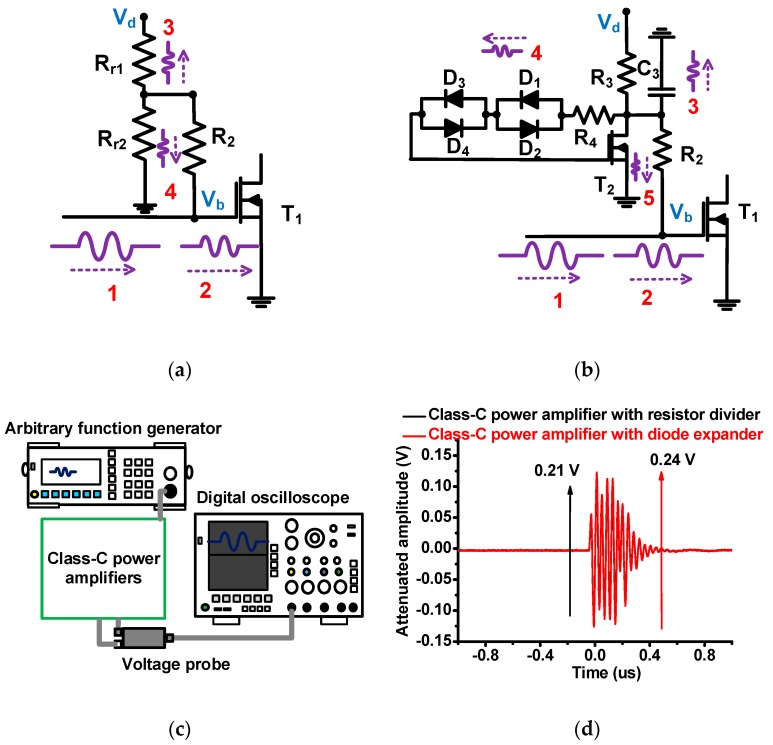
The AC operating mechanisms of the (**a**) resistor divider and (**b**) diode expander architecture; (**c**) measurement setup for input voltage attenuation in the class-C power amplifier; and the (**d**) measured AC input voltages at the gate of the LDMOSFET T_1_.

Figure 4 shows the AC operating mechanisms to predict the expected input signal paths of class-C power amplifiers with the resistor divider and diode expander architecture. As shown in Figure 4a, the pulse signals from the input port (path 1) could be transmitted to two power film resistors (R_r1_ and R_r2_, paths 3 and 4) through one power film resistor (R_2_). Likewise, Figure 4b shows that the pulse signals from the same input port (path 1) could be split into one capacitor (C_3_, path 3), one power film resistor with cross-coupled diode pairs (R_4_, D_1_ to D_4_, path 4), and one LDMOSFET (T_2_, path 5). We estimated that there are more paths to alleviate the burden into the DC power caused by unwanted AC signals. Therefore, we expect that more AC input signals could get to LDMOSFET T_1_ for a class-C power amplifier with a diode expander architecture. To confirm this idea, the input voltages at the gate of the LDMOSFET (V_b_) were measured as depicted in Figure 4c. The arbitrary function generator produced 5 V_p–p_ pulsed signals at 25 MHz for class-C power amplifiers with the resistor divider and diode expander architecture using a 10:1 voltage probe (TPP0200, Tecktronics Inc., Beaverton, OR, USA) from a digital oscilloscope (MDO4104C, Tecktronics Inc., Santa Clara, CA, USA). The measured bias voltages of the class-C power amplifier with resistor divider and diode expander architecture were 0.21 V and 0.24 V_p–p_, respectively. Therefore, we can confirm that the class-C power amplifier with a diode expander architecture would be more suitable to reduce the voltage attenuation, resulting in higher output voltage amplification.

The gain (V_G_) and DC current consumption (P_D_) of the class-C power amplifiers can be expressed [9,22] as:

(3) $$V_{G}=20\cdot {\mathrm{Log}}_{10}(i_{o}\cdot \frac{R_{L}}{2\pi \cdot V_{\mathrm{in}}}\left(2\theta_{c}-\sin2\theta_{c}\right))$$

(4) $$I_{D}=\frac{i_{o}}{\pi }\left({\sin\theta }_{c}-\theta_{c}{\cos\theta }_{c}\right)$$

Here, i_o_, R_L_, V_in_, and θ_c_ are the output current, load resistance, input voltage, and conduction angle of the class-C power amplifiers.

The gain and DC current consumption equations for class-C power amplifiers with resistor divider and diode expander architecture coincide [22]. However, the output current (i_o_) and conduction angles (θ_c_) are different given that those values are dependent on the DC bias voltages [22]. The LDMOSFET T_1_ cannot provide the expected parameter for low DC bias voltage in class-C-type power amplifiers. There are no simulation model parameters either for power film resistors (CADDOCK Electronics Inc., Roseburg, OR, USA) and LDMOSFET T_2_ in the diode expander. Therefore, modeled equations of the gain and DC current consumption are provided without simulation data. The measured performance of the class-C power amplifier with resistor divider and diode expander architecture is characterized next. 

## 3. Results and Discussion

**Figure 5:**
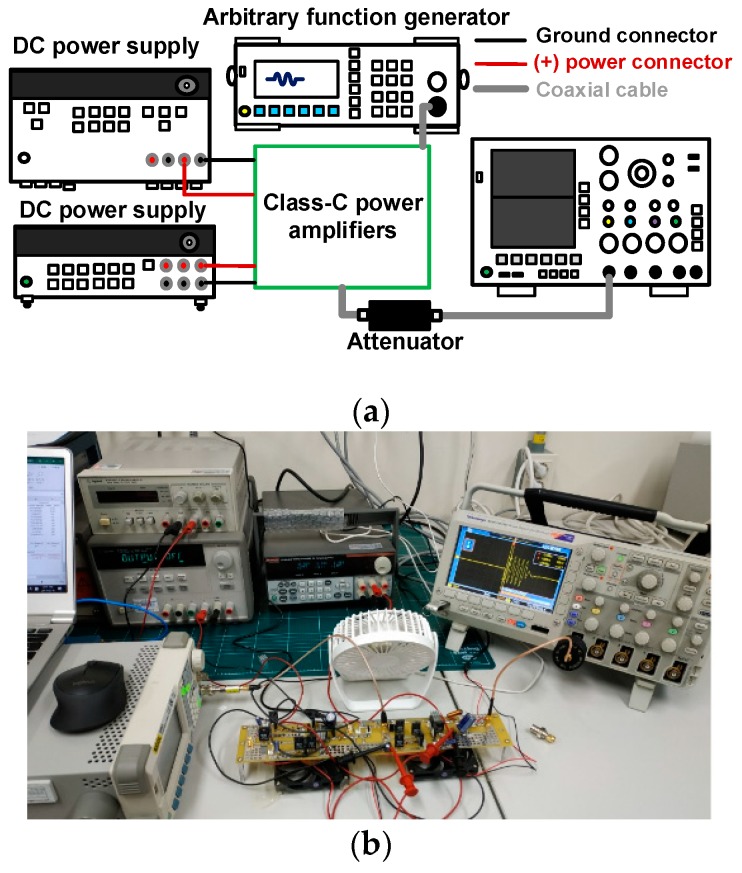

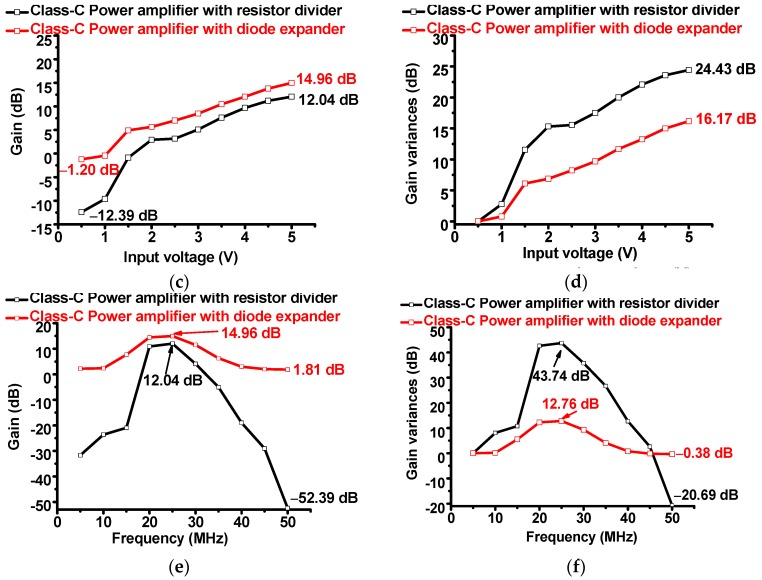
(**a**) Measurement setup sketch and (**b**) an image of its actual realization for measurement of voltage gain and DC current consumption of the power amplifiers; measured voltage gain of the class-C power amplifier versus input voltage with the (**c**) resistor divider and diode expander architecture; (**d**) measured voltage gain deviation of the class-C power amplifier versus input voltage with resistor divider and diode expander architecture; measured voltage gain of the class-C power amplifier versus frequency with the (**e**) resistor divider and (**f**) diode expander architecture.

To estimate the performance of the class-C power amplifier with a resistor divider and diode expander architecture, the measurement setup shown in Figure 5a for voltage gain versus input voltage of power amplifiers was employed. A 25 MHz five-cycle sinusoidal pulse signal with up to 5 V_p__–p_ was generated from an arbitrary function generator (DG5701, Rigol Inc., Beijing, China) and applied to the designed class-C power amplifiers to measure the output peak-to-peak voltage amplitude in a digital oscilloscope (MDO4104C). One DC power supply (2231A-3-30, Keithley Instruments, Cleveland, OH, USA) provided gate-source DC voltage and another power supply (E3647A, Agilent Technologies, Santa Clara, CA, USA) provided drain-source DC voltage for the class-C power amplifiers. The voltage gain in each case was obtained by dividing the measured output peak-to-peak voltage by the corresponding measured input peak-to-peak voltage. The pulse-echo mode is a standard method to evaluate the performance of the developed electronic components or ultrasonic transducers [23]. In the pulse-echo mode, the voltage amplitude of the echo signal generated by ultrasonic transducers is typically measured in terms of peak-to-peak voltage [24]. Therefore, voltage gain was measured through peak-to-peak voltages for ultrasound systems.

Figure 5b,c show the measured voltage gain versus input voltages of a class-C power amplifier with resistor divider and diode expander architecture. The voltage gain of the amplifier with diode expander architecture (14.96 dB) was higher than that of the amplifier with resistor architecture (12.04 dB) at 5 V_p–p_ input. Additionally, the gain deviation of the amplifier with diode expander architecture (16.17 dB) was lower than that of the amplifier with resistor architecture (24.43 dB) at 5 V_p–p_ input. Therefore, the class-C power amplifier with a diode expander architecture exhibited higher voltage gain compared to that with a resistor divider architecture. Figure 5e,f show the measured voltage gain versus frequencies of the class-C power amplifier with the resistor divider and diode expander architecture. The voltage gain of the class-C power amplifier with the diode expander architecture (1.81 dB) was higher than that of the amplifier with the resistor architecture (−52.39 dB) at 50 MHz. Additionally, gain deviation of the amplifier with diode expander architecture (−0.38 dB) is lower than that of the amplifier with resistor architecture (−20.69 dB) at 50 MHz. Therefore, the class-C power amplifier with the diode expander architecture exhibited a relatively wider bandwidth compared to that with the resistor divider architecture.

**Figure 6:**
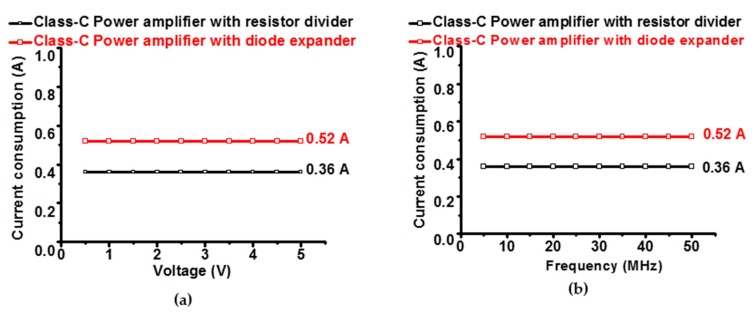
(**a**) Current consumptions of the class-C power amplifier versus input voltage and (**b**) frequency with the resistor divider and diode expander architecture.

Measured data for DC current consumption versus input voltages and frequencies of the class-C power amplifiers with the resistor divider and diode expander architecture are depicted in Figure 6a,b. In Figure 6a, a 25 MHz sinusoidal pulse signal with up to 5 V_p__–p_ was applied to the designed class-C power amplifier to measure the current consumption. In Figure 6b, a 5 V_p__–p_ sinusoidal pulse signal up to 50 MHz was applied to the designed class-C power amplifiers to measure the current consumption. The measured current consumption of the class-C power amplifier with resistor diode and diode expander architecture was less than 1 A. The measured current consumption of the amplifier with the diode expander architecture (0.52 A) was a bit higher than that of the amplifier with the resistor divider architecture (0.36 A). Therefore, both class-C power amplifiers still generated low current consumption such that it can produce relatively low heat, resulting in lower battery consumption for point-of-care ultrasound systems.

The currently designed class-C power amplifier with the diode expander architecture had a reasonable gain between 15 MHz and 35 MHz. To re-design the amplifier to cover the bandwidth between 1 MHz and 25 MHz, the input and output matching circuits need to be removed and series capacitors may be used. For such a case, the bandwidth between 1 MHz and 25 MHz could be covered in the class-C power amplifiers. However, the voltage gain could be decreased or increased as the frequency increased. To flat the bandwidth, the linearizer circuit would be helpful [25]. Therefore, the diode expander architecture needs to be replaced with another linearizer circuit. Therefore, each architecture has some trade-offs in terms of performance, such as voltage gain and bandwidth.

There is more research that needs to be done for the use of higher frequencies for a variety of applications, such as skin, eye, and intravascular imaging [23,26]. The future direction of point-of-care ultrasound systems could utilize a frequency range higher than 25 MHz, since current commercial bench-top ultrasound machines are already doing so [2]. Higher frequency operation in ultrasound machines will be more demanding due to the higher spatial resolution while scarifying the penetration depth [2].

**Figure 7:**
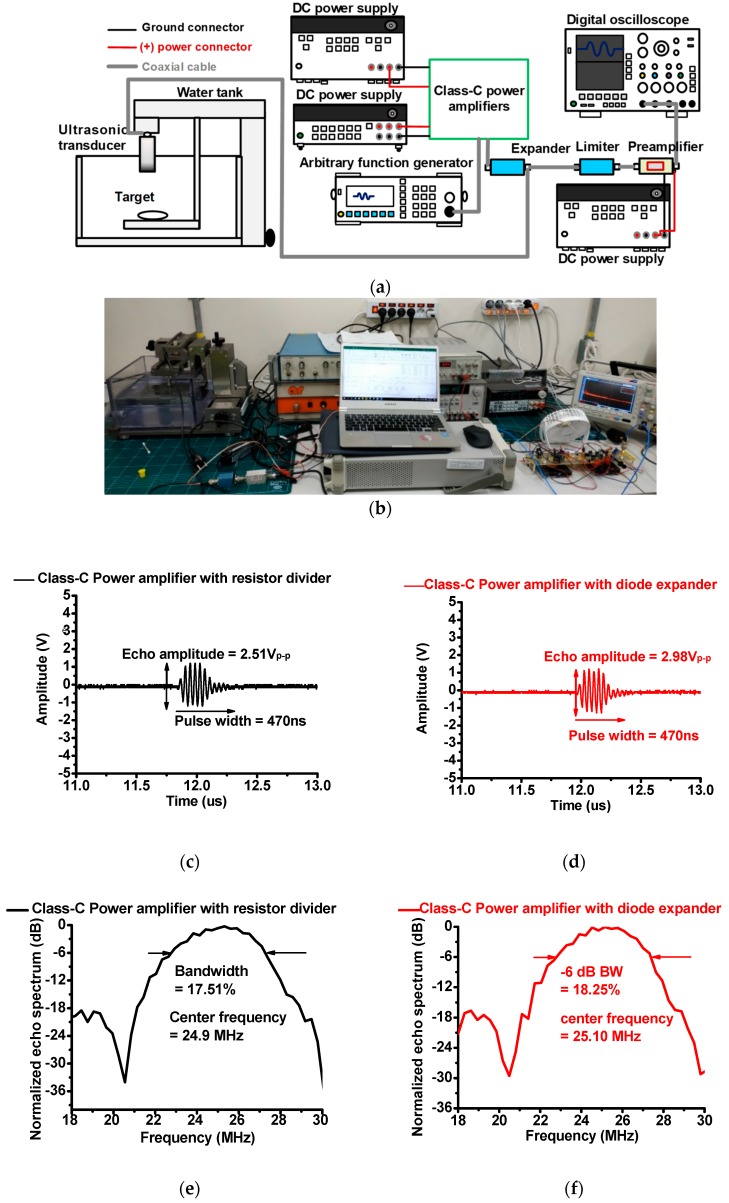
(**a**) Schematic diagram and (**b**) pictures during pulse-echo response measurement; echo signal amplitudes when using the class-C power amplifier with a (**c**) resistor divider and (**d**) diode expander architecture; echo signal spectrums when using the class-C power amplifier with a (**e**) resistor divider and (**f**) diode expander architecture.

Figure 7a shows a typical setup for pulse-echo response measurement using class-C power amplifiers with a resistor divider and diode expander architecture [18]. A 25 MHz five-cycle 5 V_p__–__p_ sinusoidal waveform from a generator (DG5701) was the input of the designed class-C power amplifiers. This input triggered the ultrasonic transducer (V324, Olympus Scientific Solutions Americas Inc., Waltham, MA, USA). Ultrasonic transducers transmitted acoustic waveforms into a circular quartz target. The reflected weak acoustic waveforms were converted into electrical waveforms by ultrasonic transducers. The resulting waveforms were then amplified by a 36 dB preamplifier (AU-1526, L3 Narda-MITEQ Inc., Hauppauge, NY, USA) to be displayed on a 1 GHz digital oscilloscope with a 5 GS/s sampling rate (MDO3102). Figure 7b,c show echo signal amplitudes and their normalized spectrum when using the class-C power amplifier with a resistor divider and diode expander architecture. The echo signal amplitude is related with the sensitivity of ultrasonic transducers [27,28,29]. In Figure 7b, echo signal amplitudes when using the class-C power amplifiers with resistor divider (2.51 V_p–p_) is a little bit smaller than that when using the amplifiers with diode expander architecture (2.98 V_p–p_). The −6 dB bandwidth is related with the image resolution of ultrasonic transducers in point-of-care ultrasound systems [30,31,32]. In Figure 7c, the echo signal spectrum when using class-C power amplifiers with a resistor divider (17.51%) was smaller than that when using the amplifiers with a diode expander architecture (18.52%). Therefore, the class-C power amplifier with a diode expander architecture outperformed the class-C power amplifier with a resistor divider architecture when it came to improving the sensitivity of point-of-care ultrasound systems.

## 4. Conclusions

In point-of-care ultrasound systems, excessive overheating critically reduces the performance of piezoelectric transducers because class-A power amplifiers in ultrasound transmitters generate unwanted heat during the entire operation. To reduce excessive overheating, non-linear power amplifiers such as class-C power amplifiers can be used given that these amplifiers have on and off transition periods during its entire operation. Because of pulse signals affecting bias circuits, class-C power amplifiers could be affected by bias circuits, resulting in the generation of sensitive outputs for ultrasonic transducers. Therefore, a new diode expander architecture dedicated to improving input signal conditions for class-C power amplifiers was proposed. The diode expander architecture could reduce the effects of unwanted input pulse signals toward bias circuits, thus reducing the attenuation of the input pulse signals for class-C power amplifiers. As a result, higher input signals could be transferred to the class-C power amplifiers. We have shown that the gain of a class-C power amplifier with a diode expander (14.96 dB) was higher than that with a resistor diode expander (12.04 dB) for a 5 V_p–p_ input. However, the current consumption of a class-C power amplifier with a diode expander architecture (1.02 W) was a little bit higher than that with a resistor divider architecture (0.75 W).

To confirm the proposed idea, typical one-way pulse-echo response measurements were taken. The echo signal amplitude and its −6 dB bandwidth when using a class-C power amplifier with diode expander architecture (2.98 V_p–p_ and 18.25%) was higher and wider than those of a class-C power amplifier with resistor divider architecture (2.51 V_p–p_ and 17.51%). The limitation of the developed class-C power amplifier with a resistor divider architecture made it difficult to block the unwanted pulse input signals. The limitation of the developed class-C power amplifier with a diode expander architecture is that the maximum voltage of the LDMOSFET in the diode expander needs to be much higher than the power supply with unwanted pulse signals. However, a class-C power amplifier with diode expander architecture can be a useful way to improve the output voltage amplitude. In the future, the developed architecture combined with a multiplexer/de-multiplexer will be applied to the array transducers because the sensitivity in point-of-care ultrasound systems is one of the critical performance issues.

## Figures and Tables

**Table 1:** Component values for class-C power amplifiers.

Components	Values	Components	Values	Components	Values	Components	Values
R_r1_	5 kΩ	C_E__2_	220 μF	C_5_	150 pF	C_6_	100 pF
R_r2_	0.5 kΩ	C_L1_	0.1 μF	C_L4_	0.1 μF	L_c_	1 μH
R_1_	56 Ω	C_L2_	1 nF	C_L5_	1 nF	L_2_	560 nH
C_E1_	10 μF	C_L3_	47 pF	C_L6_	47 pF	L_3_	220 nH
